# Supplementary material for: Bidirectional Regulation of Sodium Acetate on Macrophage Activity and Its Role in Lipid Metabolism of Hepatocytes
Source: Int J Mol Sci. 2023 Mar 14;24(6):5536. doi: 10.3390/ijms24065536 (PMC10051801; doi:10.3390/ijms24065536)
Supplement: Supplementary file 1 [file ijms-24-05536-s001.zip › ijms-2218663-supplementary.pdf]

Table S1. Sequences of primer used for RT-PCR.

Gene	Forward Primer (5′ -3′)	Reverse Primer (5′ -3′)
GAPDH	AGGTCGGTGTGAACGGATTTG	GGGGTCGTTGATGGCAACA
TNF-α	CTGAACTTCGGGGTGATCGG	GGCTTGTCACTCGAATTTTGAGA
IL-1β	TTCAGGCAGGCAGTATCACTC	GAAGGTCCACGGGAAAGACAC
IL-6	CTGCAAGAGACTTCCATCCAG	AGTGGTATAGCAGGTCTGTTGG
GPR43	GCTGACAGGCTTCGGCTTCTAC	CAGAGCAGCGATCACTCCATACAG
FAS	TATCAAGGAGGCCCATTTTGC	TGTTTCCACTTCTAAACCATGCT
Scd1	TTCTTGCGATACACTCTGGTGC	CGGGATTGAATGTTCTTGTCGT
ACC1	GATGAACCATCTCCGTTGGC	GACCCAATTATGAATCGGGAGTG
HDAC1	AGTCTGTTACTACTACGACGGG	TGAGCAGCAAATTGTGAGTCAT
HDAC2	ATACAACAGATCGCGTGATGAC	GGAACGTGAACTGCTTACCTT
HDAC3	CACCAAGAGCCTTGATGCCTT	GCAGCTCCAGGATACCAATTACT
HDAC4	CTGCAAGTGGCCCCTACAG	CTGCTCATGTTGACGCTGGA
HDAC5	AGCACCGAGGTAAAGCTGAG	GCTGTGGGAGGGAATGGTT
HDAC6	TCCACCGGCCAAGATTCTTC	CAGCACACTTCTTTCCACCAC
HDAC7	GAACTCTTGAGCCCTTGGACA	GGTGTGCTGCTACTACTGGG
HDAC8	ACTATTGCCGGAGATCCAATGT	CCTCCTAAAATCAGAGTTGCCAG
HDAC9	CAGAAGCAGCACGAGAATTTGA	CTCTCTGCGATGCCTCTCTAC
FATP2	TCCTCCAAGATGTGCGGTACT	TAGGTGAGCGTCTCGTCTCG
SREBP1c	CTTTGGCCTCGCTTTTCGG	TGGGTCCAATTAGAGCCATCTC

Table S2. Design of GPR43 siRNA targeting sequences

Gene	Forward Primer (5′ -3′)	Reverse Primer (5′ -3′)
siGPR43-1	CCUGGAUCCAUUGUUCUATT	UAGAACAAUAAGAUCCAGGTT
siGPR43-2	CCAAAUAACCUGCUACGAGAATT	UUCUCGUAGCAGAUCCAGGTT
siGPR43-3	CCGGCCACUGUAUGGAGUGAUTT	AUCACUCCAUACAGUGGCCGGTT

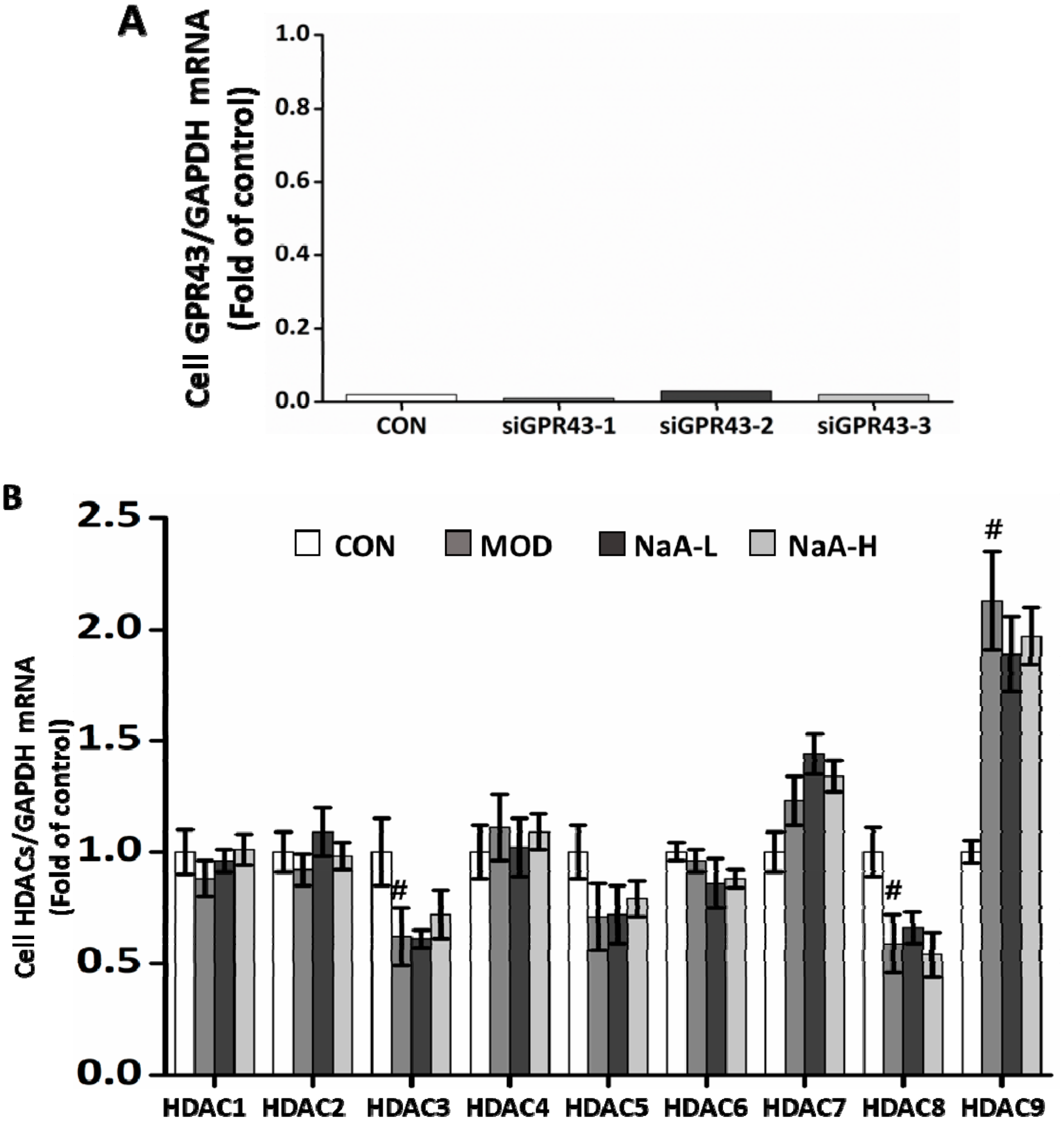

FIGURE S1 | NaA regulated macrophages inflammatory response in a GPR43/ HDACs-independent manner. (A) GPR43 was not involved in macrophages inflammatory response regulated by NaA. (B) HDACs was not involved in macrophages inflammatory response regulated by NaA. Real-time PCR was used to evaluate the mRNA expressions of HDACs. For all bar graphs, data are the mean ± SD, $\#p < 0.05$, as compared with CON. The significant difference was assessed using the one-way ANOVA followed by LSD post-tests. Control group (CON), group model cell treated with a LPS (MOD), low dose of sodium acetate group (NaA-L), high dose of sodium acetate (NaA-H), arbitrary unit (AU).

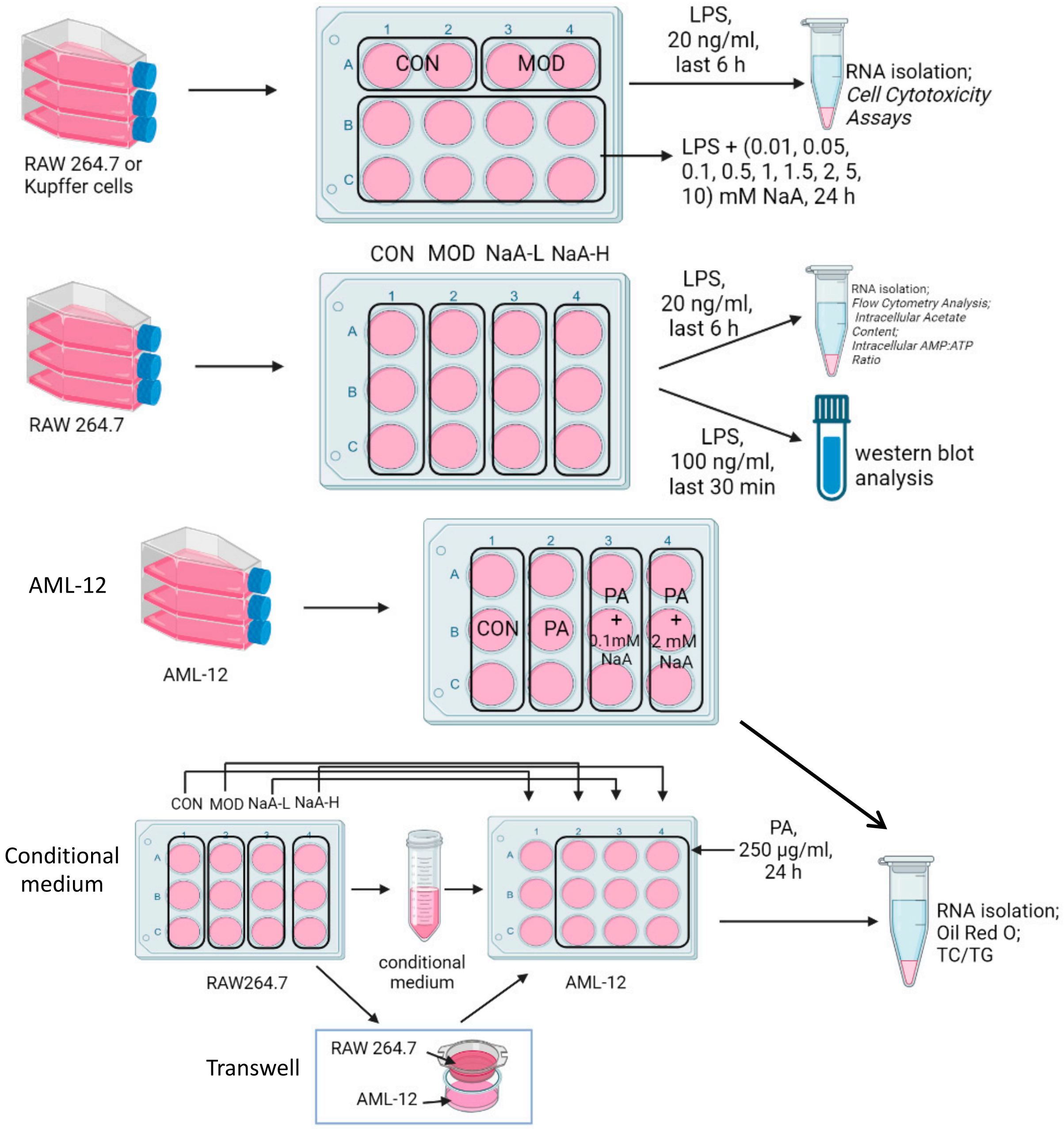

CON: contral group, macrophages (RAW264.7 /Kupffer) were incubated with fresh DMEM or RPMI 1640 medium.

MOD: macrophages (RAW264.7 /Kupffer) were incubated with DMEM or RPMI 1640 medium with LPS (100 ng/mL for the last 30 min or 20 ng/mL for the last 6 h).

NaA-L: macrophages (RAW264.7 /Kupffer) were incubated with DMEM or RPMI 1640 medium with 0.1mM of NaA for 24 h in the presence of LPS LPS (100 ng/mL for the last 30 min or 20 ng/mL for the last 6 h).

NaA-H: macrophages (RAW264.7 /Kupffer) were incubated with DMEM or RPMI 1640 medium with 2mM of NaA for 24 h in the presence of LPS LPS (100 ng/mL for the last 30 min or 20 ng/mL for the last 6 h).

FIGURE S2 丨Cell culture flow diagram
